# Nanoengineered cotton wipes for antiviral protection and environmental compatibility

**DOI:** 10.1038/s41598-025-13736-3

**Published:** 2025-08-02

**Authors:** Sunghyun Nam, Artur P. Klamczynski, Zach McCaffrey, Gregory M. Glenn, Doug J. Hinchliffe, Jonn A. Foulk, Md Nayeem Hasan Kashem, Zhongqi He, SeChin Chang, Ping Li

**Affiliations:** 1 Cotton Fiber Bioscience and Utilization Research Unit, Southern Regional Research Center, U.S. Department of Agriculture, Agricultural Research Service, New Orleans, LA 70124 USA; 2 Bioproducts Research Unit, Western Regional Research Center, U.S. Department of Agriculture, Agricultural Research Service, Albany, CA 94710 USA; 3 Office of National Programs, U.S. Department of Agriculture, Agricultural Research Service, Beltsville, MD 20705 USA; 4 Commodity Utilization Research Unit, Southern Regional Research Center, U.S. Department of Agriculture, Agricultural Research Service, New Orleans, LA 70124 USA; 5 Cotton Quality and Innovation Research Unit, Southern Regional Research Center, U.S. Department of Agriculture, Agricultural Research Service, New Orleans, LA 70124 USA

**Keywords:** Antiviral cotton wipes, Nanoengineered cotton, Mineralization, Compost, Marine environment, Materials science, Nanoscience and technology

## Abstract

Nanotechnology-based modifications enable the development of high-performance materials, expanding their applications beyond conventional uses. This study presents the production of sustainable antiviral cotton wipes through the nanoengineering of cotton fibers and investigates their mineralization behavior in compost and marine environments. Silver (Ag) nanoparticles, averaging 22 nm in diameter, were synthesized in situ using the inherent reducing agents present in raw cotton fiber and embedded within the fiber matrix. The modified cotton fibers were incorporated into nonwoven wipes using a hydroentanglement process at 20 wt%, yielding cotton wipes containing 225 mg/kg of Ag nanoparticles. The Ag-nanoengineered cotton wipes demonstrated a 99.68% reduction in virus titer against Feline calicivirus in a surface time-kill test using ready-to-use, pre-saturated wipes. Mineralization analyses indicated that both control and Ag-engineered cotton wipes followed first-order decay kinetics in compost and marine environments, with no significant difference in overall mineralization behavior. Ag-nanoengineered cotton wipes exhibited slightly lower mineralization rates, extended induction periods, and delayed maximum mineralization rates compared to control cotton wipes. Nanoengineering increased the half-life of cotton wipes by 19% in compost and 8% in marine conditions, suggesting complete mineralization within one month in compost and two months in marine environments.

## Introduction

As a renewable resource, cotton serves as a key agricultural raw material for the textile industry^1^. Its natural softness, breathability, and excellent moisture absorbency make it an ideal choice for clothing and bedding, as well as medical applications such as bandages and gauze. Additionally, its strength and durability also make it valuable for cleaning materials and filtration products^2^. However, the increasing dominance of synthetic fibers has posed significant challenges to the cotton market, particularly in the nonwoven textiles sector^3^. Nonwoven textiles, including wipes, diapers, and masks, are manufactured using processes such as hydroentangling, thermal bonding, and melt-blowing. As these products are typically designed for single-use or specialized applications, cost-efficiency and ease of production are prioritized, leading to the widespread adoption of synthetic materials^4^.

In recent years, the demand for effective and environmentally sustainable antiviral nonwoven wipes has surged, driven by concerns over infectious disease transmission and the ecological impact of synthetic materials^5^. Widely used in healthcare settings, public spaces, and households for surface disinfection, most commercially available disposable antiviral wipes are made from synthetic polypropylene and polyester^6^. While these materials reduce costs and provide durability, their extensive use has raised environmental concerns regarding microplastic pollution and long-term waste accumulation^5^.

As part of broader strategies to reduce the environmental impact of synthetic nonwoven use, efforts in the US and EU have focused on promoting the inclusion of renewable, biodegradable natural fibers in nonwoven products, especially in hygiene and personal care applications^7^. To further accelerate the transition to fully bio-based nonwovens, it is crucial to develop technologies that enhance the performance of natural fibers, making them competitive with synthetic fibers. One promising strategy involves the use of nanotechnology-based modifications, which can introduce new functionalities to fibers^8^. However, the current application of nanotechnology remains costly for disposable nonwoven products^9^. The synthesis of nanoparticles typically requires expensive chemical agents, such as reducing agents to convert metal ions to metal atoms and stabilizing agents to prevent particle aggregation. Additionally, techniques are needed to control particle growth, along with specialized machinery to ensure uniform nanoparticle application^10^. These technical and economic challenges pose significant barriers to integrating nanotechnology into high-performance, sustainable nonwoven products.

This study presents a sustainable approach to nanoengineering cotton fibers for the development of antiviral nonwoven wipes. Rather than using pre-synthesized Ag nanoparticles—which are costly due to purification, stabilization, and handling requirements—we employed an in situ synthesis method that utilizes raw cotton fibers as natural reducing and stabilizing agents^11–15^. This bio-based process integrates Ag nanoparticles directly within the fiber matrix, eliminating the need for external chemical additives and post-synthesis treatments. The resulting Ag-nanoengineered cotton fibers were incorporated in the fabrication of hydroentangled nonwoven wipes and evaluated for their antiviral performance and environmental sustainability. To assess biodegradability, the mineralization kinetics of both Ag-engineered and control cotton wipes were examined in compost and marine environments. This study demonstrates the dual functionality of cotton as both a fiber structural substrate and a platform for bio-enabled nanoparticle synthesis, offering a cost-effective and environmentally responsible alternative to synthetic fiber-based wipes.

## Materials and methods

### Materials

Mechanically cleaned raw cotton fibers were obtained from Wildwood Cotton Technologies (Greenwood, MS, USA). Silver nitrate (AgNO_3_, 99.9%) was purchased from J. T. Baker (Radnor, PA, USA). Triton X-100, methyl methacrylate, butyl methacrylate, benzoyl peroxide, and nitric acid (HNO_3_, 70%, Trace Metal Grade) were obtained from Sigma-Aldrich (St. Louis, MO, USA). All chemicals were used as received. Deionized (DI) water was used as a solvent for all preparations.

## Methods

### Preparation of Ag-nanoengineered cotton wipes

Approximately 35 g of cotton fibers were immersed in 500 mL of an aqueous solution containing 0.05 wt% Triton X-100 for 5 min. The wetted fibers were then centrifuged using a spin dryer to remove the excess solution. Subsequently, the fibers were immersed in 500 mL of a 1 mM AgNO_3_ aqueous solution and heated at 100 °C for 1 h. The treated fibers were then centrifuged and air-dried.

To fabricate Ag-nanoengineered cotton wipes, the treated cotton fibers were blended with control cotton fibers at a 20/80 wt% ratio, then carded and hydroentangled at the nonwoven pilot plant of the Southern Regional Research Center in New Orleans, LA, following a published procedure^16^. The resulting hydroentangled cotton wipes had an area density of 65 ± 6 g/m^2^. Cotton wipes made of control cotton fibers were also fabricated for comparison.

### Characterization

Optical microscopy images were obtained in reflection mode using a digital microscope (KH-8700, Hirox, Oradell, NJ, USA). The 3D optical profile analysis was conducted using a digital microscope (VHX-7000, Keyence, Itasca, IL, USA). TEM images and selected area electron diffraction (SAED) were obtained using a TEM (JEM-2011, Jeol, Peabody, MA, USA) operating at 200 kV. Fiber cross-sections for TEM were prepared following published methods^17,18^ and placed on copper grids coated with a carbon film. The size of Ag nanoparticles was measured from TEM images using Image-J software^19^.

The Ag content in the cotton fibers was determined using a graphite furnace atomic absorption spectrometer (240Z AA, Agilent, Santa Clara, CA, USA). For sample preparation, approximately 0.05 g of fibers was digested with 10 mL of 6 M HNO_3_ in a microwave digestion system (MARS 6, CEM Corporation, Matthews, NC, USA). The resulting digest solution was diluted at a ratio of 1:1000 by weight and analyzed using an external calibration curve generated with an Ag single-element standard (Agilent, Santa Clara, CA, USA). The reported Ag concentration represents the average of three measurements.

Ultraviolet-visible (UV-vis) spectra were obtained using a UV-vis spectrometer (ISR-2600, Shimadzu, Columbia, MD, USA) equipped with an integrating sphere unit. Diffuse reflectance spectra were collected across the 200–1400 nm wavelength range.

### Virucidal efficacy test

The virucidal efficacy of ready-to-use, pre-saturated cotton wipes against Feline calicivirus (F-9 strain, ATCC VR-782) was evaluated using three replicates following ASTM E1053 at Microchem Laboratory (Round Rock, TX, USA). The Ag-nanoengineered cotton wipes were pre-saturated in DI water at a 2:1 liquor ratio. A sterile glass Petri dish (15 cm × 2 cm) was inoculated with 0.2 mL of virus suspension at 22 °C and 46% relative humidity and dried at 20 °C and 30% relative humidity for 22 min. The wiping procedure consisted of three passes, with one pass defined as a single back-and-forth motion. After wiping, the treated carriers were left for a 10-min contact time at room temperature (22 °C and 46% relative humidity). At the end of the exposure period, 2.0 mL of test media was pipetted onto the carrier surface to neutralize the test sample, and the viral films were resuspended. The collected suspension was then passed through a gel filtration column. A plate recovery control was prepared identically, except without wiping. The neutralized viral suspensions were quantified using a tissue culture infective dose (TCID_50_) assay. Inoculated cell culture plates were incubated at 36 °C with 6% CO_2_ for approximately seven days, after which viral cytopathic effects were evaluated microscopically. Virus titers and cytotoxicity were determined, and the log_10_ and percent reductions in viral titer were calculated using the following equations:

1 $$\:{Log}_{10}\:reduction={log}_{10}\left({TCID}_{50}\:of\:control\right)-{log}_{10}\left({TCID}_{50}\:of\:test\:sample\right)$$

2 $$\:Percent\:reduction=1-\left(\frac{C}{B}\right)\times\:100$$

where *B* is the average TCID_50_ of the virus in control suspensions, and *C* is the average TCID_50_ in test sample suspensions. Any test sample cytotoxicity was accounted for in the calculations. Quantification was performed with a minimum of four determinations per dilution.

### Mineralization study

Percent mineralization was determined by dividing the measured CO_2_ volume by the theoretical CO_2_ yield. The measurement of CO_2_ volume was conducted using an automated respirometer system (Microoxymax, Columbus Instruments, Columbus, OH, USA) equipped with a CO_2_ sensor ranging 0–3%, following ASTM D6691-17. The theoretical CO_2_ yield was calculated based on the carbon content and moisture content of the samples. Starch was used as a positive control. The carbon content of each sample was determined using an elemental combustion analyzer (Vario EL cube, Elementar, Analysensysteme GmbH, Hesse, Germany) by combusting 2 mg of the sample at 1150 °C. The measured carbon contents were 43.9% for starch, 41.5% for cotton, and 41.5% for Ag-cotton. Moisture content was determined by drying samples overnight at 105 °C. The measured moisture contents were 6.37% for cotton and 5.88% for Ag-cotton.

### Mineralization in compost

Mineralization in compost was evaluated following ASTM D5338-03 with modifications. Compost was purchased from a local hardware store, sieved through a 1 mm screen, and a fraction smaller than 1 mm was used. Moisture content was determined by drying the compost overnight at 105 °C. During the experiment, compost moisture was maintained between 57 and 58%. Cotton samples (cut into 1 cm × 1 cm squares) were placed in 1 L medium bottles and mixed with 40 g of compost pre-hydrated to 57.5% moisture. The initial sample weight ranged from 0.37 to 0.5 g. Bottles were connected to the automated respirometer to monitor CO_2_ evolution. The experiment was conducted at 58.5 °C. Throughout the experiment, samples were mixed twice a week using a spatula to ensure good contact between the compost and the sample. Water loss was monitored, and moisture was adjusted as needed to maintain 57–58% compost moisture. All experiments were performed in triplicate.

### Mineralization in the marine environment

Mineralization in marine conditions was evaluated following the ASTM D6691-17 method with modifications. Seawater was collected from the open California coast and allowed to settle for 24 h. After settling, the clear seawater was carefully siphoned from above the sediment. The water was supplemented with 0.5% NH_4_Cl and 0.1% KH_2_PO_4_ as specified in ASTM D6691-17.h Cotton samples weighing 0.5 g were placed in 1 L media bottles containing 500 mL of the fortified seawater. The bottles were connected to the automated respirator to monitor CO_2_ evolution (in microliters). The temperature was maintained at 30 °C, and the bottles were placed on orbital shakers to ensure continuous water movement. All experiments were conducted in triplicate.

### Mineralization kinetics

The percent mineralization data were converted to the percent carbon remaining and analyzed using a first-order decay model, which assumes that the rate of biodegradation is proportional to the remaining carbon:

3 $$\:{C}_{t}={C}_{0}{e}^{-kt}$$

where *C*_*t*_ is the carbon remaining at time *t* (%), *C*_*0*_ is the initial carbon content (%), i.e., the carbon at time *t* = 0, *k* is the first-order decay rate constant (day^−1^), and *t* is time (days). To determine the rate constant *k*, which characterizes the mineralization rate, the natural logarithm was applied to both sides of the equation:

4 $$\:{lnC}_{t}={lnC}_{0}-kt$$

A linear regression was performed on the plot of *lnC*_t_ versus *t*, where the slope (-*k*) provides the rate constant. The half-life (*t*_1/2_) representing the time required for 50% biodegradation, was calculated as:

5 $$\:{t}_{1/2}=\frac{{ln}2}{k}$$

To determine whether there was a significant difference in mineralization rates, an F-test was performed. The delta method was used to estimate the standard errors for the decay rate constant and half-life^20^.

## Results and discussion

### Ag nanoengineering of cotton fibers

**Fig. 1:**
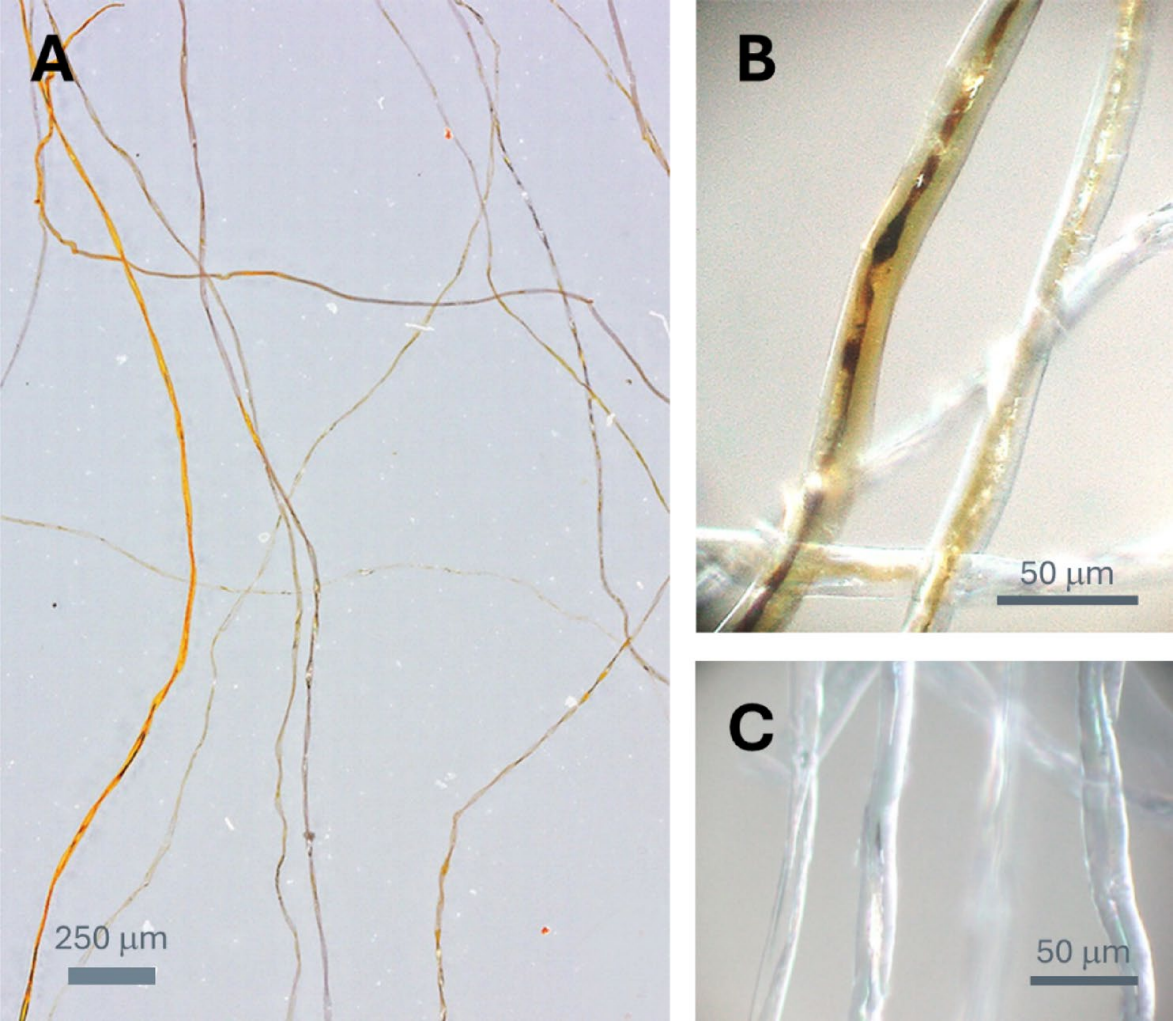
Optical microscopy images of Ag-nanoengineered cotton fibers at (**A**) low and (**B**) high magnifications. (**C**) Optical microscopy image of control cotton fibers at high magnification.

Figure 1A shows an optical microscopy image of the longitudinal view of Ag-nanoengineered cotton fibers. The incorporation of Ag nanoparticles within the cotton fibers altered their coloration, producing hues ranging from orange to dark brown. This color change is attributed to the surface plasmon resonance (SPR) of Ag nanoparticles. SPR occurs when the free conduction electrons on the nanoparticle surface interact with the electromagnetic field of incident light^21^. This interaction results in distinct absorption and scattering, generating vibrant colors. The shade and intensity of the color depend on factors such as particle size, shape, concentration, and the material matrix in which the nanoparticles are embedded^22^.

The observed variations in hue and shade among fibers suggest differences in their synthetic capacity. These differences arise from the in situ synthesis of Ag nanoparticles being reliant solely on the natural reducing agents present in raw cotton fibers, such as sugars, pectin, cell nuclei, protoplasm, and other metabolic byproducts. The presence of these non-cellulosic components varies between individual fibers, even within the same plant^23^. Fibers with higher concentrations of these non-cellulosic components exhibit increased reducing properties and subsequently synthesize greater quantities of Ag nanoparticles.

Higher magnification images were obtained to investigate the in situ formation of Ag nanoparticles within the cotton fiber microstructure, which were compared with images of untreated control cotton fibers (Fig. 1B and C, respectively). Cotton fibers are around 20 μm thick and exhibit a flattened, ribbon-like morphology. This characteristic morphology is the result of the protoplasm within the fiber’s central cavity (lumen) drying out^24^. Comparing the longitudinal views of treated and untreated fibers revealed that the lumen area of treated fibers displayed more pronounced coloration, indicative of increased Ag nanoparticle formation. Natural reducing agents, including protoplasm and metabolic byproducts concentrated in the lumen, facilitated the in situ synthesis of Ag nanoparticles. While some microscopic variability was observed due to the natural heterogeneity of cotton fibers, the overall loading of Ag nanoparticles is expected to be relatively consistent across fibers. This is because mature cotton fibers possess similar levels of non-cellulosic components that serve as intrinsic reducing agents. Therefore, fibers of comparable maturity and growth origin are likely to yield uniform nanoparticle formation under identical reaction conditions. Under the synthesis conditions employed in this study, the Ag nanoparticle production was quantified at 1,275 ± 32 mg/kg based on the dry weight of the fibers.

**Fig. 2:**
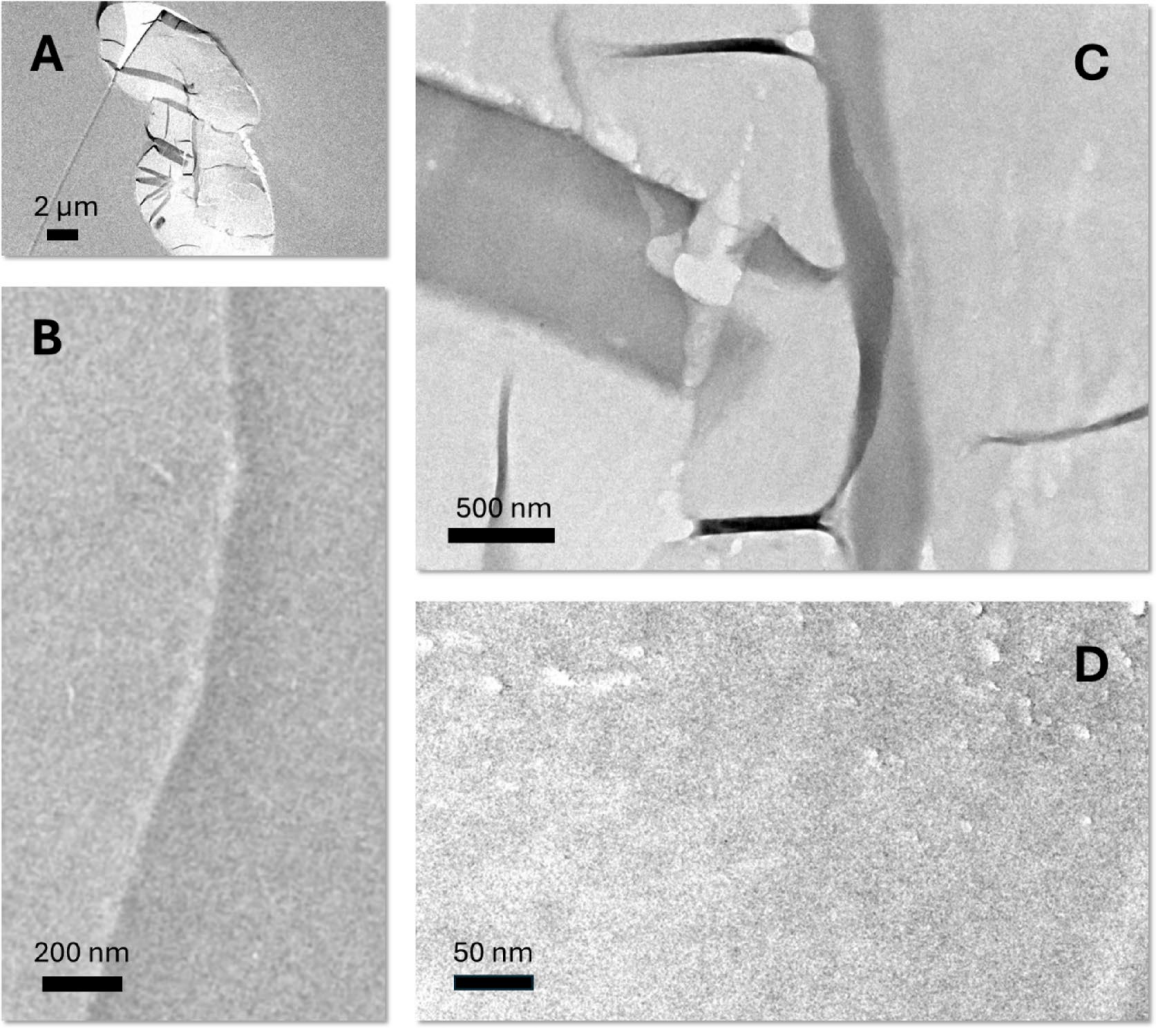
TEM images of the cross-section of control cotton fiber: (**A**) low-magnification image, (**B**) high-magnification image of the edge region, and (**C**, **D**) high-magnification images of the central region.

To investigate how Ag nanoparticles were formed across the fiber diameter, cross-sectional views of control and treated cotton fibers were examined by TEM. Figure 2 presents TEM images of control cotton fibers at various regions and magnifications. Cotton fibers have a multilayered structure including the cuticle, primary cell wall, secondary cell wall, and lumen. The cuticle and primary cell wall, which form the outer layers, contain non-cellulosic components, whereas the secondary cell wall consists of cellulose^24^. The lumen contains residual protoplasm and metabolic byproducts. Due to the low electron density of cellulose and non-cellulosic components, the multilayer structural details were not distinctly visible under TEM without the application of staining or other contrast-enhancing methods.

**Fig. 3:**
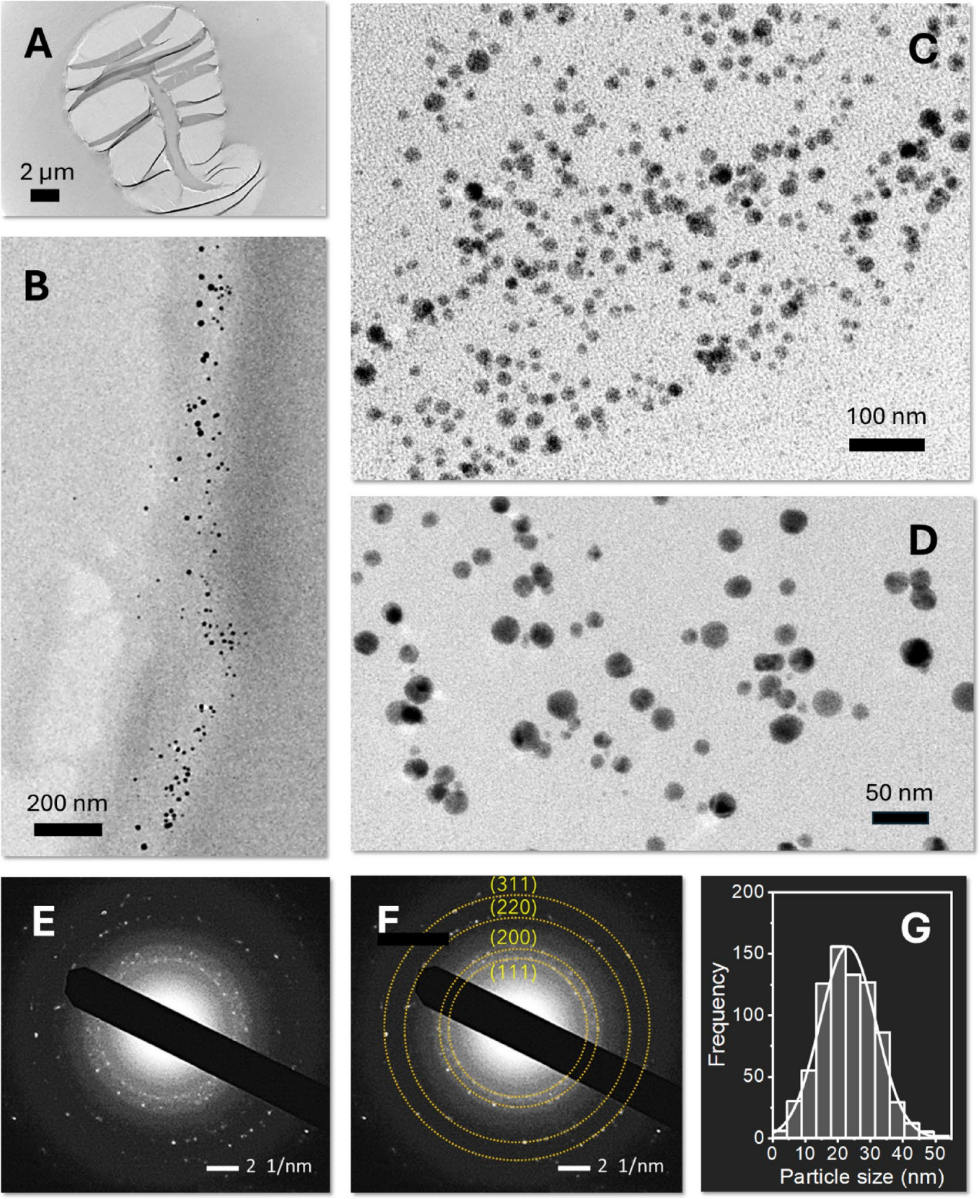
TEM images of the cross-section of Ag-nanoengineered cotton fiber: (**A**) low magnification image, (**B**) high magnification image of the edge region, (**C**, **D**) high magnification images of the central region. SAED patterns of Ag-nanoengineered cotton fiber (**E**) without and (**F**) with marked rings indicating the diffraction planes of Ag nanoparticles. (**G**) Histogram of the Ag nanoparticle size distribution, with the solid line representing a Gaussian fit.

The low contrast of the cotton fiber under TEM allowed the clear visualization of Ag nanoparticles formed within the fiber matrix. Figure 3A and D show TEM images of Ag-nanoengineered cotton fibers at low magnification (Fig. 3A), at high magnification of the fiber’s outer layer (Fig. 3B), and high magnifications of the central region (lumen) (Fig. 3C and D). The images reveal that Ag nanoparticles are predominantly formed in the outer layers and lumen, confirming that natural reducing agents enabled the reduction reactions of Ag ions. The nanoparticles exhibit near-spherical morphology and are mostly individually dispersed, indicating that the in situ synthesis of nanoparticles within cotton fibers does not require stabilizing agents.

The SAED analysis validated that the particles embedded in the fiber are composed of elemental Ag, This is evidenced by four distinct concentric diffraction rings corresponding to the (1 1 1), (2 0 0), (2 2 0), and (3 1 1) planes of the face-centered cubic (fcc) crystalline structure of Ag (Fig. 3E and F). The weak diffraction spots likely result from the small size of the nanoparticles within the cotton fiber matrix. Figure 3G shows a histogram of the nanoparticle size distribution, fitted to a Gaussian function. From multiple TEM images, the average diameter of the Ag nanoparticles was determined to be 22.7 ± 7.5 nm.

**Fig. 4:**
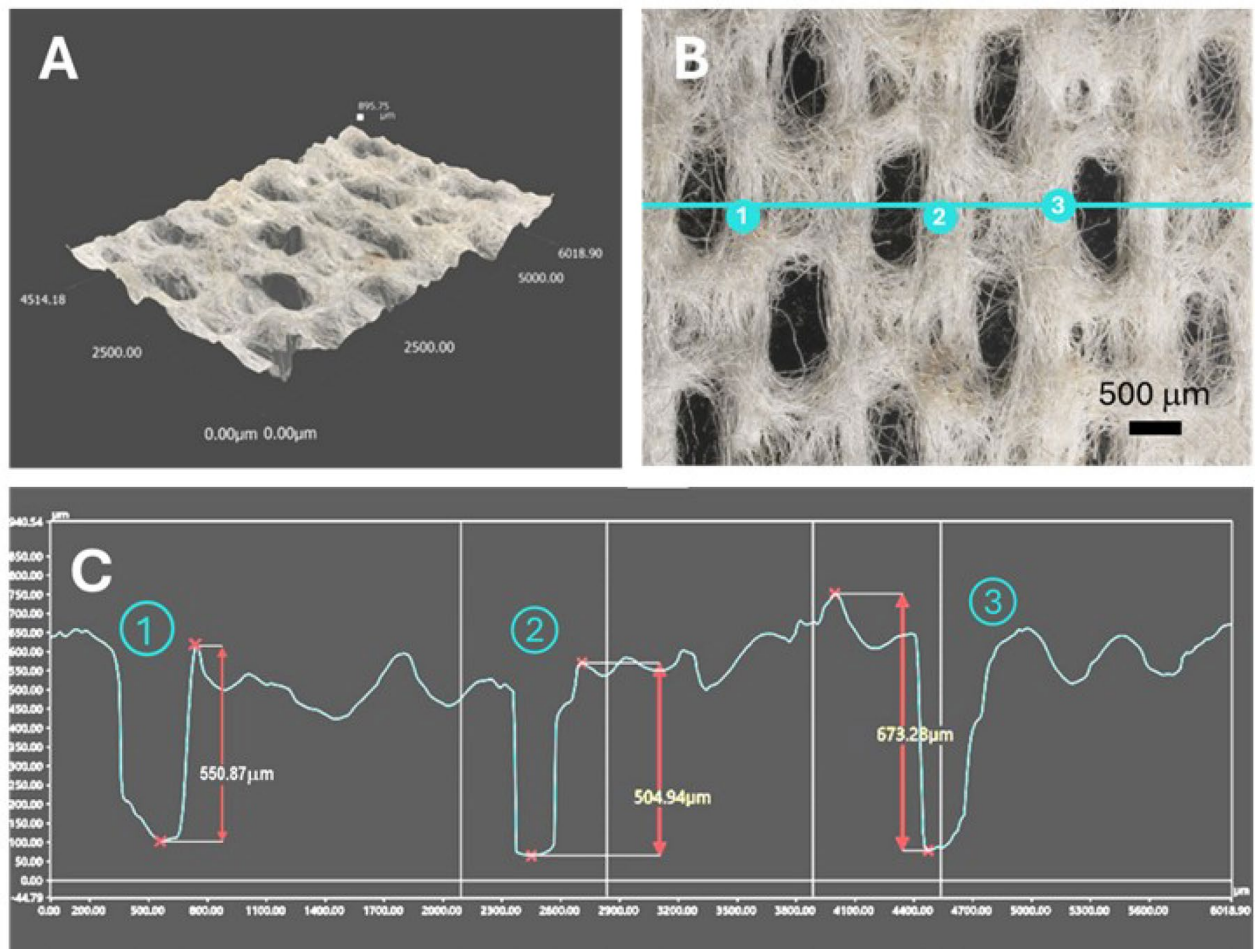
(**A**) 3D optical profile, (**B**) optical microscopy image, and (**C**) microstructure analysis of a hydroentangled nonwoven wipe fabricated from a 20 wt% blend of Ag nanoengineered cotton fibers.

### Ag-engineered cotton wipes

Ag-engineered cotton nonwoven wipes were fabricated using a hydroentanglement process in a pilot plant. In this process, 20 wt% Ag-nanoengineered cotton fibers were uniformly blended with 80 wt% control cotton fibers through three rounds of carding. The resultant blended fiber web underwent hydroentanglement. For comparison purposes, control nonwoven wipes were fabricated from control cotton fibers alone. The Ag concentration in the resultant wipes was 225 ± 18 mg/kg. Figure 4 shows the microstructure of the resulting wipes, as analyzed through 3D optical profiling. The thickness of the fabricated wipes was approximately 0.6 mm. The hydroentanglement process effectively interlocked the fibers, producing a cohesive fabric structure. High-pressure water jets (9 MPa) penetrated the fiber web, which created localized perforations. These perforations were elongated, likely due to the motion of the web during hydroentanglement, which caused stretching along the direction of movement. The perforations exhibited relatively uniform size and distribution, indicating the efficiency of the hydroentanglement process in producing consistent nonwoven fabrics while maintaining structural integrity from short staple cotton fibers. Previous studies^25–27^ have demonstrated that hydroentangled nonwoven fabrics made from cotton fibers exhibited mechanical and physical properties comparable to those of competitive nonwoven substrates, supporting their mechanical robustness and functional durability. It was also found that embedding Ag nanoparticles into cotton fibers reinforced the fibers, slightly improving their tensile strength and toughness^28^. Together, these results indicate the suitability of Ag-engineered cotton hydroentangled nonwoven fabrics for use in wipe applications.

### Antiviral cotton wipes

**Fig. 5:**
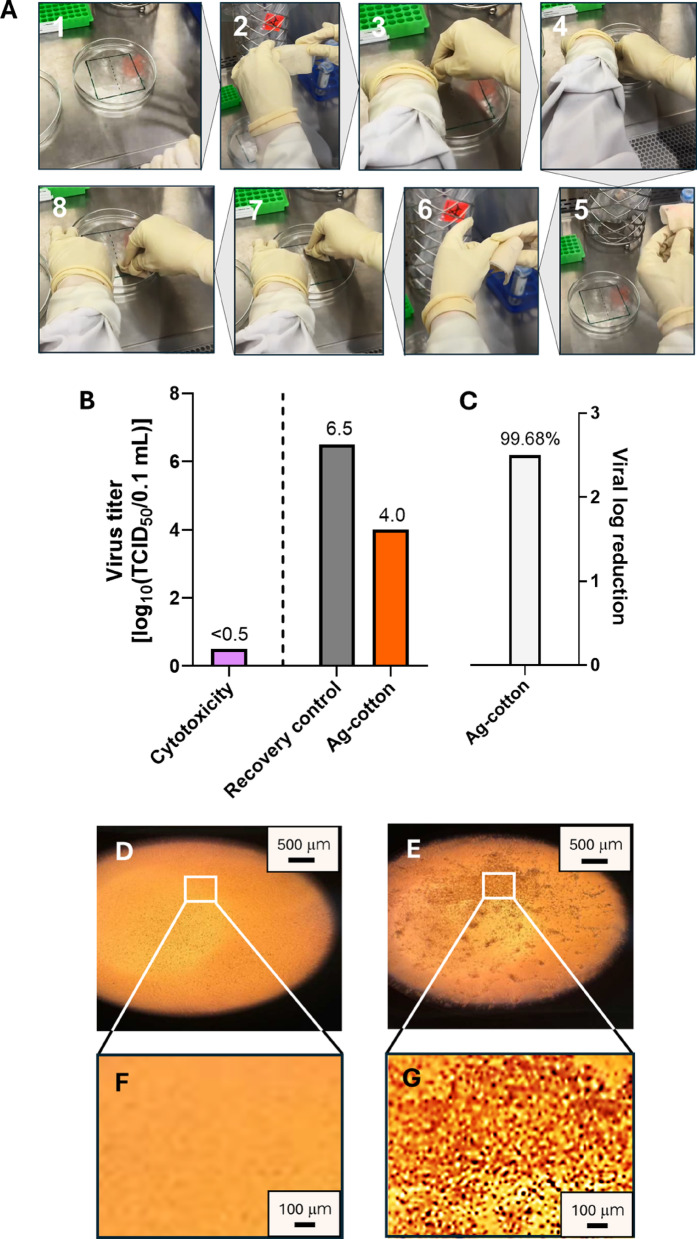
(**A**) Photographs of the wiping procedure in the virucidal efficacy test of ready-to-use, pre-saturated Ag-nanoengineered cotton wipes. (**B**) Cytotoxicity assessment of Ag-engineered cotton wipes and the titers of Feline calicivirus for the recovery control plate and the plate wiped with Ag-engineered cotton wipes. (**C**) Log reduction of Feline calicivirus after wiping with Ag-engineered cotton wipes. The number atop the bar represents the percent reduction. Optical microscopy images of (**D**) a healthy cell monolayer and (**E**) a Feline calicivirus-infected cell monolayer at 40x magnification. The boxed areas in (**D**) and (**E**) are magnified in (**F**) and (**G**), respectively, at 4x magnification.

The antiviral efficacy of Ag-engineered cotton wipes against Feline calicivirus was evaluated using a standardized surface time-killed test following ASTM E1053, a method designed to simulate real-world disinfection scenarios on hard, nonporous environmental surfaces. The wipes were tested in their final, ready-to-use, pre-saturated form, which reflects the typical application format of disinfectant wipes during practical use. The Ag-engineered cotton wipes were wetted in DI water. The wiping procedure is shown in Fig. 5(A). Each wipe (18 cm × 19 cm) was folded longitudinally four times, creating a final size of approximately 5 cm × 5 cm. The folded wipe was further shaped into a “U” configuration around the index finger for application. The virus film on the carrier surface was divided into two equal sections (1). The first section was wiped with three back-and-forth passes (six total motions) (2–4). The wipe was then refolded to expose a fresh, unused surface (5, 6), which was used to wipe the second section of the carrier using the same procedure (7, 8).

Figures 5(B) and 5(C) show the results of the surface time-kill test (ASTM E1053). The cytotoxicity assessment, performed using the TCID_50_ assay, shows that the Ag-engineered cotton wipes exhibit a cytotoxic effect of less than 0.5 log_10_ TCID_50_ per 0.1 mL. This minimal cytotoxicity suggests that the wipes have low toxicity toward the tested cell line. Figures 5(D)−5(G) show optical microscopy images comparing a healthy cell monolayer with a cell monolayer infected with Feline calicivirus. The healthy cell monolayer appears structurally intact with a smooth, homogeneous texture. In contrast, the infected cell monolayer exhibits pronounced cytopathic effects, characterized by cell weakening, lysis, and loss of structural integrity. Ag-nanoengineered cotton wipes reduced virus titers from 6.5 ± 0.37 log_10_ TCID_50_ per 0.1 mL to 4.0 ± 0.19 log_10_ TCID_50_ per 0.1 mL, representing a 2.5 log_10_ reduction or 99.68% virus inactivation (*p* < 0.01). This antiviral activity is attributed to the action of Ag^+^ ions, which interact with viral DNA or RNA and inhibit replication^29^. Moisture from the pre-saturation process promotes oxidative dissolution of silver from the surface of the embedded nanoparticles, resulting in the release of Ag^+^ ions onto the wipe surface. Ion release is important in both the functional performance and environmental behavior of metallic nanomaterials. Previous studies have shown that Ag^+^ release from Ag nanoparticles reaches equilibrium between 24 and 48 h^30–32^. In our system, the embedded nanoparticles act as a reservoir, releasing Ag^+^ ions upon pre-saturation, thereby charging the wipe surface with antiviral functionality prior to use. During application, the released Ag^+^ ions come into direct contact with viral particles. These ions interact with thiol, carboxyl, and amine groups in viral proteins and nucleic acids, leading to structural disruption and loss of infectivity^29^.

### Mineralization in compost and marine environments

**Fig. 6:**
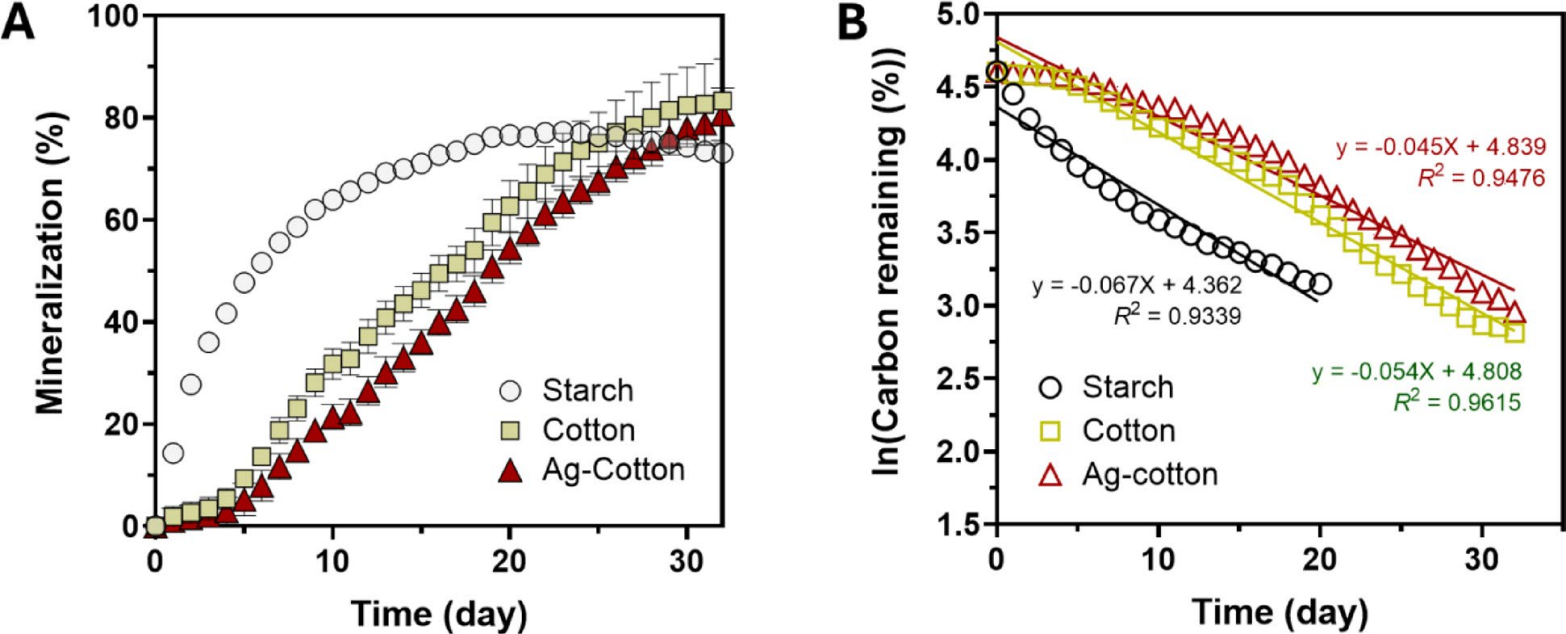
(**A**) Percent mineralization of starch, control cotton wipes, and Ag-engineered cotton wipes as a function of days in compost. (**B**) Natural logarithm of the percent carbon remaining as a function of days in compost. Straight lines represent regression analysis using the first-order decay model. The starch data up to 20 days were used for regression analysis.

Figure 6A shows the percent mineralization of starch, control cotton wipes, and Ag-engineered cotton wipes in compost. Starch, used as a positive control to validate experimental and theoretical CO_2_ volumes, exhibited the highest mineralization rate, with rapid mineralization observed during the first 10 days. After this initial phase, the rate declined, reaching a plateau of approximately 77% by day 20. For control cotton wipes and Ag-engineered cotton wipes, both required several days to initiate mineralization and subsequently mineralized at a slower rate compared to starch, with a slight deceleration starting around day 25. Throughout the period studied, the average percent mineralization of Ag-engineered cotton wipes was slightly lower than that of control cotton wipes. To further examine the mineralization kinetics in compost, a first-order decay model was applied. For starch, the data prior to reaching the plateau were analyzed. As shown in Fig. 6B, plotting the natural logarithms of the percent carbon remaining against time produced linear correlations for starch, control cotton wipes, and Ag-engineered cotton wipes. Regression analysis yielded coefficients of determination (*R*^2^ greater than 0.9 for all three samples, indicating first-order decay kinetics. According to the F-test, the first-order decay kinetics for control cotton wipes and Ag-nanoengineered cotton wipes were not significantly different (*p* = 0.8250).

**Fig. 7:**
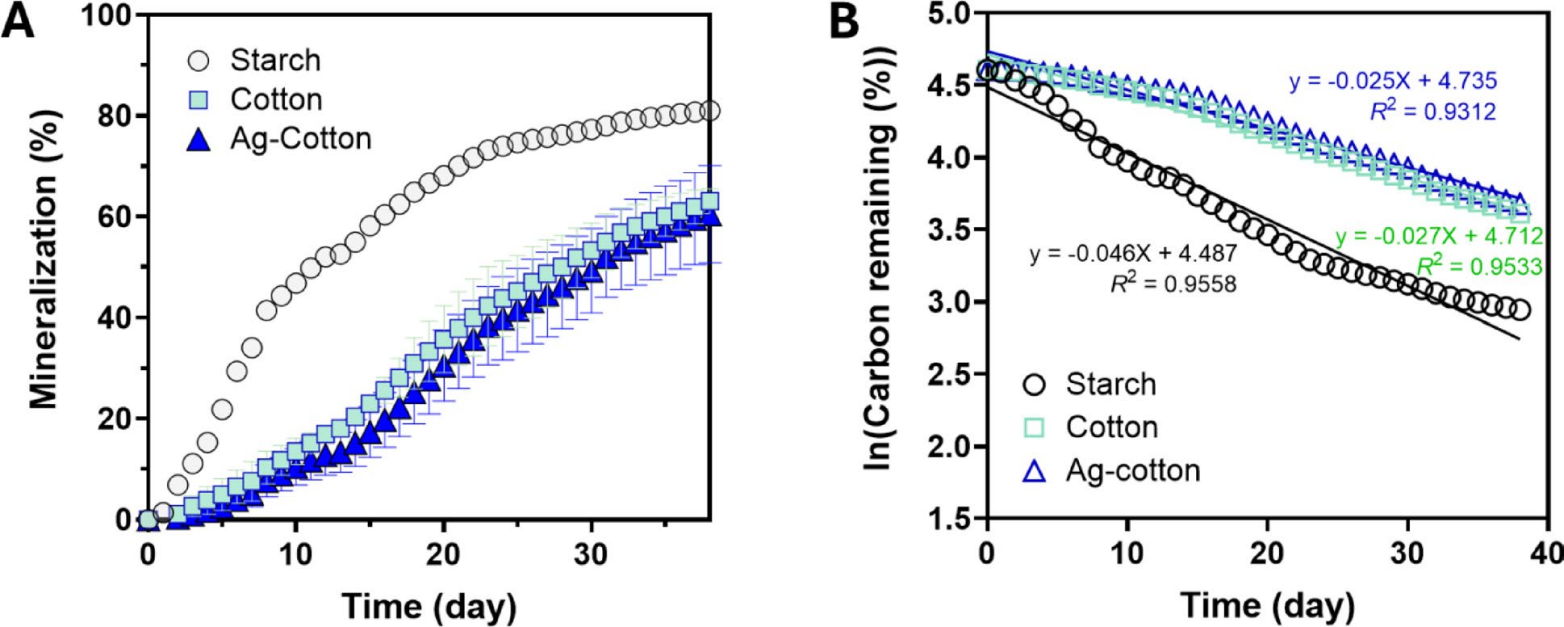
(**A**) Percent mineralization of starch, control cotton wipes, and Ag-engineered cotton wipes as a function of days in the marine environment. (**B**) Natural logarithm of the percent carbon remaining as a function of days in the marine environment. Straight lines represent regression analysis using the first-order decay model.

Figure 7A shows the percent mineralization of starch, control cotton wipes, and Ag-engineered cotton wipes in a marine environment. In comparison to compost, all samples mineralized at a slower rate, with mineralization continuing throughout the period studied. Consistent with the compost results, starch exhibited the fastest mineralization. The average percent mineralization of Ag-engineered cotton wipes remained lower than that of control cotton wipes, similar to the trend observed in compost. However, the difference between their rates was smaller in the marine environment. The plot of the natural logarithm of the percent carbon remaining versus time (Fig. 7B) also yielded linear trends, confirming that the mineralization in the marine environment adheres to first-order decay kinetics. Notably, the slopes of these trends were shallower than those observed in compost. As expected, an F-test comparing the first-order decay kinetics of control cotton wipes and Ag-nanoengineered cotton wipes produced a higher *p*-value (0.9958).

**Fig. 8:**
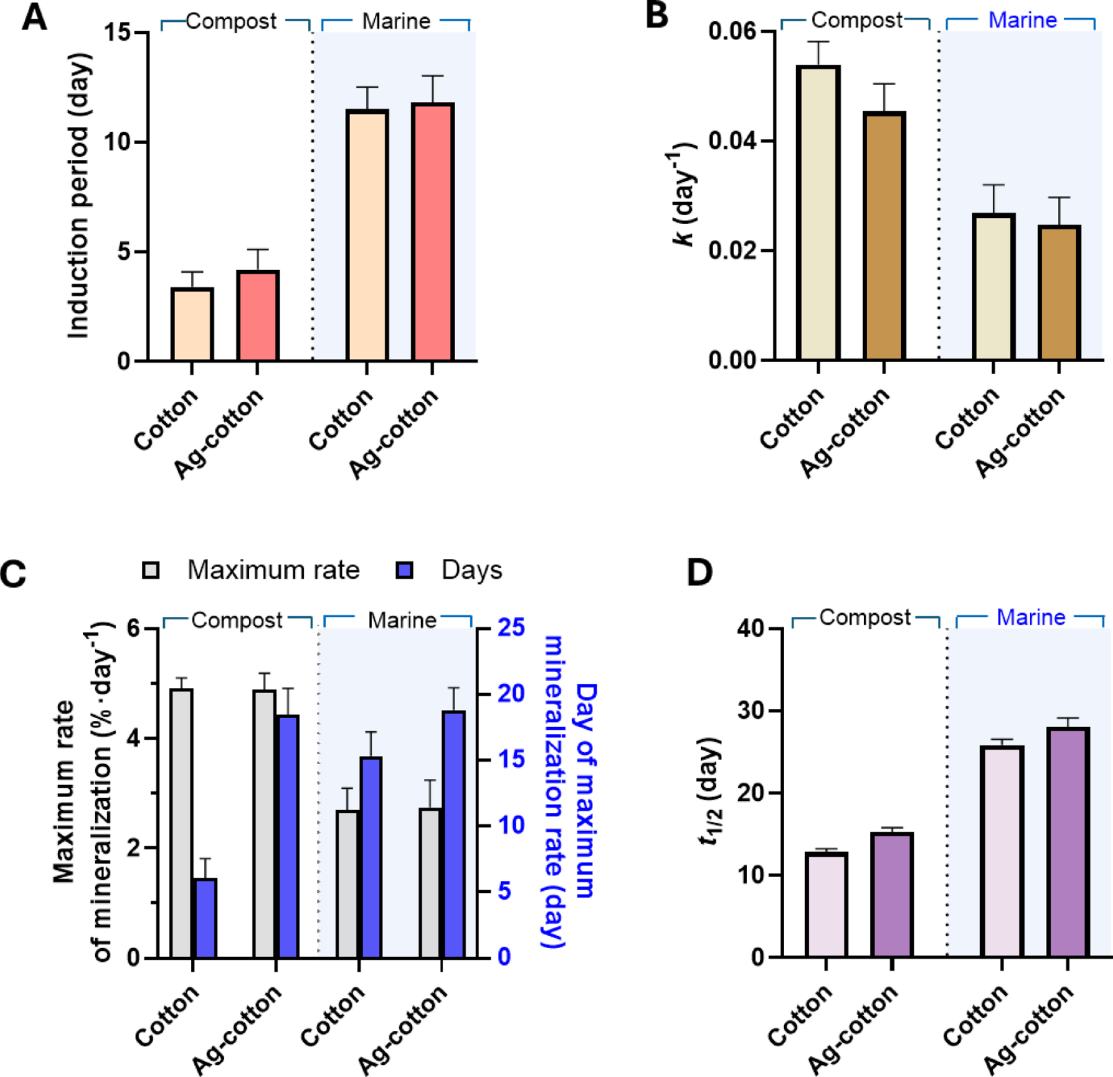
(**A**) Induction periods, (**B**) rate constants, (**C**) maximum mineralization rates and their corresponding days of occurrence, and (**D**) half-lives of mineralization for control cotton wipes and Ag-engineered cotton wipes in compost and marine environments.

Mineralization kinetic parameters for control cotton wipes and Ag-engineered cotton wipes in compost and marine environments were compared. Figure 8A shows the induction period, which represents the time required before significant mineralization begins. This period was determined by identifying the time before a strong linear correlation (*R*^2^ > 0.9) in the fit of the first-order decay model. In the compost environment, the induction period for Ag-engineered cotton wipes was slightly longer than that of control cotton wipes (*p* < 0.05), indicating that Ag-engineered cotton wipes exhibited slightly higher resistance to mineralization. The release of Ag ions from the Ag nanoparticles in the moist compost environment may inhibit microbial activity, thus extending the induction period. Previous studies^11–13,33^ have demonstrated that Ag-nanoengineered cotton fabrics inhibited representative Gram-positive and Gram-negative bacteria as well as a soil-borne fungus. In the marine environment, the induction periods for both wipes were approximately three times greater than those observed in compost. The longer induction period in the marine environment can be attributed to slower microbial activity, higher salinity, and reduced nutrient availability, all of which hinder microbial mineralization. The difference in induction period between control cotton wipes and Ag-engineered cotton wipes was smaller in the marine environment. This reduced difference is likely due to the overall slower and less efficient microbial mineralization under marine conditions.

Figure 8B shows the rate constants of control cotton wipes and Ag-engineered cotton wipes in compost and marine environments. Although the F-tests on the first-order decay kinetics did not indicate a statistically significant difference, it was observed that the nanoengineering of cotton wipes with a low concentration of Ag nanoparticles slightly reduced the rate constant, with the effect more pronounced in the compost environment. The compost environment, which supports higher microbial activity, promotes faster mineralization, resulting in higher rate constants for both types of wipes. The marine environment, characterized by reduced microbial activity, led to lower overall rate constants. This slower mineralization process under marine conditions likely accounts for the smaller difference observed between the rate constants of control cotton wipes and Ag-engineered cotton wipes.

The maximum mineralization rate was determined by calculating the derivative of percent mineralization over time during the most rapid phase. Figure 8C shows the maximum mineralization rate and the corresponding day of occurrence for control cotton wipes and Ag-engineered cotton wipes in compost and marine environments. Consistent with other measured parameters, the maximum mineralization rates were higher in compost than in the marine environment, likely due to the environmental factors discussed earlier. Within the same environment, no significant difference was observed in the maximum mineralization rates between control cotton wipes and Ag-engineered cotton wipes. However, the time required to reach the maximum mineralization rate was significantly longer for Ag-engineered cotton wipes compared to control cotton wipes (*p* < 0.05). While the compost environment expedited the occurrence of the maximum mineralization rate for control cotton wipes, this effect was not observed for Ag-engineered cotton wipes. The resistance of Ag-engineered cotton wipes to microbial activity in compost may delay the onset of rapid mineralization.

Figure 8D shows the half-life, calculated using Eq. (5), for control cotton wipes and Ag-engineered cotton wipes in compost and marine environments. Nanoengineering of cotton wipes resulted in a slight increase in the half-life in compost, rising from 12.9 days to 15.3 days, an increase of approximately 19%. In the marine environment, the half-life increased from 25.9 days to 28.1 days, an increase of approximately 8%. When the environment shifted from compost to marine conditions, the half-life of control cotton wipes exhibited a twofold increase, whereas Ag-engineered cotton wipes showed an 84% increase. Ag-engineered cotton wipes are expected to fully mineralize within one month in compost environments and within two months in marine environments.

## Conclusion

This study demonstrates the advancement of cotton’s utility through nanoengineering, resulting in antiviral cotton wipes with minimal toxicity and environmental impact. The antiviral functionality was built into cotton fibers through in situ synthesis of Ag nanoparticles within the fiber matrix, using their natural reducing agents. The modified cotton fibers (20 wt%) were blended with pristine cotton fibers and fabricated into hydroentangled nonwoven wipes, yielding a final Ag concentration of 225 mg/kg. The Ag-engineered cotton wipes exhibited potent antiviral activity, achieving a 99.68% reduction in Feline calicivirus on contaminated surfaces while maintaining low cytotoxicity in tested cell lines. Environmental assessments revealed only slight differences in mineralization kinetics of Ag-engineered cotton wipes, indicating minimal overall impact on biodegradation. Both Ag-engineered and control cotton wipes followed first-order decay kinetics in compost and marine environments. The estimated half-lives showed a modest delay in complete mineralization: from 12.9 to 15.3 days in compost and from 25.9 to 28.1 days in the marine environment.

The ability of Ag-nanoengineered cotton wipes to combine antiviral efficacy with demonstrated environmental compatibility suggests their potential as a sustainable and functional alternative to synthetic fiber-based wipes. This design is also expected to offer cost-effectiveness across four key areas: (1) In situ nanoparticle synthesis using naturally occurring reducing agents within the cotton matrix eliminates the need for external chemical reductants, stabilizers, and post-processing steps; (2) Built-in antiviral functionality enables the wipes to function as self-activating antiviral materials rather than passive carriers of externally applied antiviral agents—eliminating the need to pre-saturate wipes with excess volumes of disinfectant solutions; (3) Efficient silver utilization enabled by a high surface-area-to-volume ratio and optimized interfacial contact delivers potent antiviral activity at a low Ag loading; and (4) Embedded nature of Ag nanoparticles within the cotton fiber matrix offers the potential for extended antiviral efficacy beyond single use. Together, these features suggest that Ag-engineered cotton wipes could be a valuable addition to hygiene product development and sustainable innovation in the cotton industry.

## Data Availability

The raw data of this study are available from the corresponding author upon request.
